# Role of the nervous system in cancer metastasis

**DOI:** 10.1186/s13046-018-0674-x

**Published:** 2018-01-15

**Authors:** Nyanbol Kuol, Lily Stojanovska, Vasso Apostolopoulos, Kulmira Nurgali

**Affiliations:** 1 Centre for Chronic Disease, College of Health and Biomedicine, Victoria University, Melbourne, Australia; 2 Department of Medicine, Western Health, The University of Melbourne, Regenerative Medicine and Stem Cells Program, AIMSS, Melbourne, Australia

**Keywords:** Neurotransmitters, Neuropeptides, Neuro-cancer interaction, Metastasis, Cancer

## Abstract

Cancer remains as one of the leading cause of death worldwide. The development of cancer involves an intricate process, wherein many identified and unidentified factors play a role. Although most studies have focused on the genetic abnormalities which initiate and promote cancer, there is overwhelming evidence that tumors interact within their environment by direct cell-to-cell contact and with signaling molecules, suggesting that cancer cells can influence their microenvironment and bidirectionally communicate with other systems. However, only in recent years the role of the nervous system has been recognized as a major contributor to cancer development and metastasis. The nervous system governs functional activities of many organs, and, as tumors are not independent organs within an organism, this system is integrally involved in tumor growth and progression.

## Background

Cancer is the leading cause of death worldwide due to the aging population and unhealthy lifestyle [1]. Although it is highly treatable when localized, metastatic or recurrent cancer has a poor prognosis. Metastasis involves a complex series of steps including proliferation, angiogenesis, embolization, dissemination, evasion of immune system surveillance and surviving in ectopic organs [2–5]. However, despite significant advances in understanding metastasis and its mechanisms, the prognosis remains poor. In the past decades, research has focused on identifying and characterising genes and gene products that manipulate the metastatic processes [6–9]. More recently, the impact of the tumor microenvironment on tumor cell invasion and metastasis has attracted extensive attention (see ref. [10] for detailed review) [2, 10–13]. Multiple cellular and extracellular components within the tumor microenvironment, such as immune cells, endothelial cells, mesenchymal stromal cells (fibroblasts and myofibroblasts), and their secretory products, exert active functions to modulate gene expression patterns of tumor cells and to alter biological behavior of tumor cells [14–16]. Invariable crosstalk amongst these components within the tumor microenvironment triggers pro-survival, invasion, and metastatic pathways of tumor cells [17–20]. Several studies, both clinical and in vitro, reinforce the concept of the nervous system involvement in cancer metastasis [5, 21–26]. Nerve fibers present in and around the tumor could release neurotransmitters and neuropeptides directly acting on receptors expressed by cancer cells. The findings, primarily in cancer cell lines and animal models, indicate that there is a bi-directional correlation between the neural factors released and cancer progression and metastasis. Understanding the complex neurotransmitter-cancer interaction is important for the development of new avenues for targeted therapeutic intervention. This review presents an overview of the role of the nervous system in cancer metastasis.

## The role of the nervous system in metastatic cascade

Studies have demonstrated that the nervous system facilitates development of tumor metastasis by modulating metastatic cascades through the release of neural-related factors from nerve endings such as neurotrophins, neurotransmitters and neuropeptides [27–29]. The process of metastasis formation involves tumor cells breaking away from the primary tumor and overcoming the obstacles of primary tissue inhibition (initiation and clonal expansion), anoikis inhibition (evasion from apoptosis), breakdown of base membranes (epithelial-mesenchymal transition (EMT) and invasion), extravasation and colonization, angiogenesis, evasion of immune response and establishment of tumor microenvironment.

### Initiation and clonal expansion

Tumor metastasis initiation and clonal expansion is a complex process where contributing factors are not well understood. It is believed that metastasis process is initiated when genetically unstable tumor cells adjust to a secondary site microenvironment [11]. This process involves selecting traits that are beneficial to tumor cells and affiliated recruitment of traits in the tumor stroma that accommodate invasion by metastatic cells. Metastasis-initiating cells possess these traits and can hijack some of the normal stem cell pathways to increase cellular plasticity and stemness [30]. Proteolytic enzymes such as matrix metalloproteinases (MMPs) facilitate this process by degrading the surrounding normal tissues. MMPs are regulated by neural-related factors and neurotransmitters and are overexpressed in tumors [31–35]. Hence, nervous system modulates the initiation and clonal expansion via the expression of MMPs and the stimulation of metastasis-initiating cells.

### Evasion from apoptosis

Anoikis is a programmed cell death induced upon cell detachment from extracellular matrix, acting as a critical mechanism in preventing adherent-independent cell growth and attachment to unsuitable matrix, thus avoiding colonizing of distant organs [36, 37]. For tumor metastasis to progress, tumor cells must be resistant to anoikis. Tumor cell resistance to anoikis is attributed to alteration in integrins’ repertoire, overexpression of growth factor receptor, activation of oncogene, activation of pro-survival signals, or upregulation/mutation of key enzymes involved in integrin or growth factor receptor signaling [37]. Neurotransmitters and neurotrophins play a role in tumor evasion from anoikis. Increased expression of brain-derived neurotrophic factor (BDNF) and its receptor tropomyosin-related kinase B (TrkB) induces anoikis inhibition in rat intestinal epithelial cells [27]. Similarly, TrkB overexpression induces anoikis inhibition protecting colorectal cancer cells [38]. Application of recombinant human BDNF to gastric cancer cells inhibited anoikis and stimulated cellular proliferation, invasion and migration [39]. Nicotine exposure promotes anchorage-independent growth of A549, MDA-MB-468 and MCF-7 cell lines by downregulation of anoikis [40]. Furthermore, tumor microenvironment contributes to anoikis resistance of cancer cells by producing pro-survival soluble factors, triggering EMT, enhancing oxidative stress, regulating matrix stiffness, as well as leading to metabolic deregulations of cancer cells [37]. These events assist tumor cells to prevent the apoptosis mechanism and sustain pro-survival signals after detachment, counteracting anoikis.

### EMT and invasion

EMT is a fundamental process for tumor progression by increasing invasiveness and resistance to anoikis and significantly elevating the production of extracellular matrix constituents leading to metastasis [41–43]. EMT development results in the degradation of basement membrane and formation of mesenchymal-like cells [42]. Studies have demonstrated that nervous system regulates EMT development via the release of neurotransmitters and neurotrophins [40, 44]. The overexpression of TrkB or activation by BDNF in human endometrial cancer cell lines results in altered expression of EMT molecular mediators [44]. Nicotine treatment induces changes in gene expression associated with EMT in lung and breast cancer cells [40].

### Extravasation and colonization

Nervous system modulates the function of vascular system which is essential for tumor cell extravasation and colonization. It has been found that neuropeptides such as substance P (SP) and bradykinin enhance vascular permeability promoting tumor cell extravasation and colonization [28, 29]. In a mouse model bearing sarcoma 180 cells, bradykinin enhances tumor-associated vascular permeability [28]. SP regulates physiological functions of vascular system including smooth muscle contractility, and vascular permeability [29]. Cell extravasation and colonization are prerequisite for angiogenesis which is a crucial step in the development of cancer metastasis.

### Angiogenesis

Development of tumor angiogenesis is essential for tumor growth and progression. Vascular endothelial growth factor (VEGF) plays significant role in tumor angiogenesis, leading to metastasis [45–47]. Studies have demonstrated the important role of neurotransmitters and neuropeptides in regulating angiogenesis. In the xenograft models of ovarian cancer, chronic stress mediates the vascularization of intraperitoneal metastasis and enhances tumor angiogenesis via increasing VEGF expression [48, 49]. In breast cancer cell lines, direct activation of β-adrenergic signaling can amplify expression of VEGF and cytokines, interleukin (IL)-6, and IL-8 that stimulate tumor angiogenesis [50]. In colon tumor tissues from HT-29 cell-bearing BALB/c mice, VEGF expression is elevated by nicotine which correlates with enhanced microvessel density [51]. Neuropeptide Y (NPY) enhances the expression of VEGF and its secretion promoting angiogenesis and breast cancer progression [52].

### Evasion of immune response

The nervous system plays a fundamental role in regulating immune responses [53]. Inflammatory mediators can activate sensory nerves that send signals regarding inflammation to the central nervous system, which in turn leads to the release of neuromediators modulating local inflammation and influencing immune cells [54]. Since inflammatory signals are important for tumor progression in both the early and late stages, the anti-inflammatory role of the vagus nerve may play an important role in cancer metastasis [55]. β-adrenergic receptor agonist suppressed natural killer (NK) cell activity resulting in increased lung metastasis in murine metastatic mammary adenocarcinoma [56]. In addition, pharmacological or stress-associated β-adrenergic stimulation results in increased macrophage infiltration and cancer metastasis in breast cancer model [57].

### Tumor microenvironment

Tumor microenvironment (mainly contain stromal cells and signal molecules) plays essential role in the formation of cancer metastasis. Stromal cells produce neural-related factors and express β-adrenergic receptor that facilitated tumor cell proliferation and survival in the primary site and secondary organ [10, 24]. Tumor-associated macrophages play a role in β-adrenergic signaling pathways, by accelerating angiogenesis, chemokine secretion to attract tumor cells, secretion of pro-inflammatory cytokines (IL-1, IL-6, IL-8, and tumor necrosis factor (TNF)-α) and escape of anti-tumor responses [58–60]. Hence, tumor microenvironment creates a feedback loop with the nervous system enabling the growth of primary and secondary tumors. Overall, these studies have demonstrated that the nervous system modulates each step of cancer metastasis through the release of neural-related factors.

## Role of perineural invasion in cancer metastasis

Perineural invasion (PNI) also known as neurotropic carcinomatous spread is a process mainly categorized by neoplastic invasion of the nerves. PNI is defined as the presence of cancer cells in the perineurium; it is believed to be a common route for cancer metastasis can cause cancer-related pain [61–68]. The presence of PNI is mostly associated with poor prognosis and high recurrence in colorectal [69], gastric [64], oral tongue squamous cell carcinoma (OTSCC) [62], and pancreatic [61] cancers. In stage II and III colorectal cancer patients, the presence of PNI is associated with tumor grade, metastasis to lymph nodes and poor patient survival [63]. However, in invasive breast carcinoma the presence of PNI has been demonstrated to have no prognostic value [67, 70].

PNI is influenced by the interaction between the nerve microenvironment and neurotrophic molecules expressed by cancer cells such as nerve growth factor (NGF), BDNF, glial cell line-derived neurotrophic factor (GDNF) and their receptors [61, 68, 71]. A number of studies demonstrated correlation between the presence of PNI with high expression of NGF and its receptor tropomyosin related kinase A (TrkA) [61, 72, 73]. It is speculated that neurotrophins released by neural tissue act as chemotactic factors, and in cancer cells where Trks are overexpressed, they provide mechanism to invade the perineural space. High expression of NGF or TrkA and P75^NTR^ receptors is associated with lymph node metastasis in a mouse model of breast cancer [74]. In OTSCC patients [73], the presence of PNI and NGF is associated with larger tumor size and lymph node metastasis, suggesting that its presence can be a valuable marker to predict the disease progression and prognosis [65]. Overexpression of TrkA associates with enhanced growth, invasion and migration of breast cancer cells in vitro as well as enhanced metastasis in xenografted immunodeficient mice via the PI3K-AKT and ERK/P38 MAP kinases [75]. Conversely, immuno-histochemical evaluation of tissues from patients with extrahepatic cholangiocarcinoma shows that intra-tumoral NGF expression does not correlate with PNI, absence of disease recurrence and overall patient survival [76]. GDNF has been demonstrated to induce cancer cells migration. In human pancreatic adenocarcinoma tissues and MiaPaCa-2 cell lines, binding of GDNF to its receptor GFRα1 stimulates PNI via GDNF-(Ret proto-oncogene) RET signaling pathway [71]. Activation of GDNF-GFRα1-RET signaling triggers the MAPK signaling pathway leading to pancreatic cancer cell migration toward nerves in both in vitro and animal models of PNI [77]. Cancer-nerve interaction studied in in vitro co-cultures of DRG and MiaPaCa-2 pancreatic cancer cells demonstrated that GFRα1 facilitates migration of cancer cells along neurites toward the center of the DRG [71]. Furthermore, decreased release of soluble GFRα1 from DRG inhibits migration of cancer cells towards nerves in vivo providing further evidence that GFRα1 expression is important in facilitating PNI [71]. In a metastatic breast cancer model, in vivo inhibition of Ret suppresses tumour outgrowth and metastatic potential [78].

BDNF facilitates cancer metastasis via binding to its receptors, TrkB/ TrkC and/or p75NTR as demonstrated in breast [79], colorectal [80, 81], clear cell renal cell carcinoma [82] and non-small cell lung cancer (NSCLC) [83]. The expression of TrkB associates with nodal metastasis and peritoneal metastasis; whereas, TrkC expression associates with liver metastasis in colorectal cancer patients [81]. BDNF-TrkB signaling pathway mediates metastatic effect through modulation of cancer-associated fibroblasts (CAFs) as demonstrated in mouse model co-injected with OSC19-Luc transfected cell line and CAFs [84]. In melanoma, neurotrophin (NT)-3, NT-4, and NGF induce cell migration, with a stronger effect on metastatic cell lines via binding to p75NTR coreceptor sortilin [85]. In breast cancer, NT-3 enhances breast cancer metastasis in the brain via promoting the mesenchymal–epithelial transition of breast cancer cells to a more epithelial-like phenotype and via increasing the ability of these cells to proliferate in the brain [86].

Collectively, these studies demonstrate that neurotrophins and their receptors play crucial role in PNI. These studies also suggest that the presence of PNI could be an effective predictor of metastatic potential and patient survival.

## Tumor innervation influencing cancer metastasis

### Tumor innervation

Cancer-related neurogenesis (tumor innervation) is attributed to the ability of cancer cells to attract normal nerve fibers via the secretion of signalling molecules and neurotrophic factor. However, recent study has demonstrated that cancer stem cells are capable of directly initiating tumor neurogenesis [87]. Cancer stem cells derived from human gastric and colorectal cancer patients generate neurons including sympathetic and parasympathetic neurons which promote tumor progression [87]. Knocking down their neural cell generating abilities inhibit tumor growth in human xenograft mouse model. Neurogenesis and its putative regulatory mechanisms have been reported in prostate [88], gastric [89], colorectal [90] and breast [91] cancers. There is a correlation between the expression of a pan-neuronal marker protein gene product 9.5 with clinicopathological characteristics of breast cancer [91]. In fact, neurogenesis is associated with aggressive features including tumor grade, poor survival as well as angiogenesis, especially in estrogen receptor-negative and node-negative breast cancer subtypes [91, 92]. In prostate cancer, infiltration of the tumor microenvironment by nerve fibers associates with poor clinical outcomes [93] and is driven by the expression of granulocyte colony-stimulating factor (G-CSF) [94] and proNGF [95]. Similarly, in orthotopic PC3-luc xenografts model of prostate cancer, neurogenesis and axonogenesis correlate with aggressive features including metastatic spread which is attributed to the neo-cholinergic parasympathetic nerve fiber [94]. These findings indicate that neurogenesis, like angiogenesis, is also a trait of cancer invasion and can alter tumor behaviour.

### Tumor denervation

On the other hand, disruption of tissue innervation might cause accelerated tumor growth and metastasis [56, 96–101]. For instance, in humans, decreased vagal nerve activity correlates with advanced stages of cancer [96–98]. Similarly, modulation of vagal nerve activity enhances metastasis of breast cancer in mice [99, 100]. In addition, capsaicin-induced inactivation of sensory neurons enhances metastasis of breast cancer cells [56, 101]. On contrary, pharmacological or surgical denervation supresses the tumor progression as noted in three independent mice models of gastric cancer [89]. Thus, these findings suggest that there might be differences in the effects of local tumor innervation and extrinsic innervation on cancer progression.

## Neurotransmitters influencing cancer metastasis

**Fig. 1:**
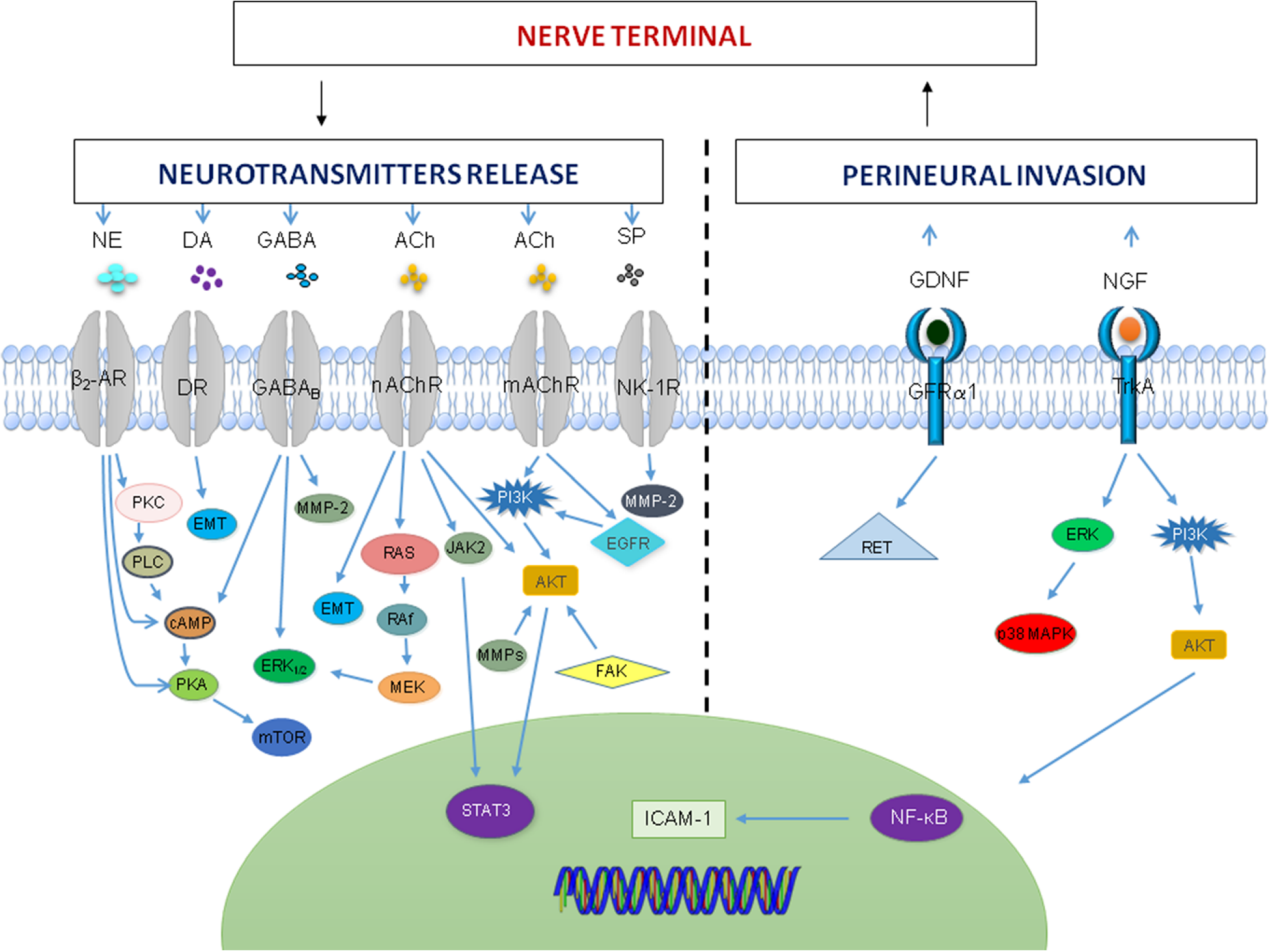
Neurotransmitters signalling pathways in cancer. Cancer neuro-immune communication is through the release of neurotransmitters using different signalling kinases which promote cancer progression via metastasis. Perineural invasion mediate cancer metastasis through the release of the NGF and GDNF via the activation of different signaling pathway. Ach, acetylcholine; β2-AR, β_2_-adrenergic receptor;cAMP, cyclic adenosine monophosphate; DA, dopamine; DR, dopamine receptor; EGFR, epidermal growth factor receptor;EMT,epithelial–mesenchymal transition; ERK_1/2_, extracellular signal-regulated kinase;FAK, focal adhesion kinase; GABA, gamma-aminobutyric acid; GABA_B_,gamma-aminobutyric acid receptor_B_;GDNF, glial cell line-derived neurotrophic factor; GFRα, glial cell line-derived neurotrophic factor receptor 1;ICAM-1, intercellular adhesion molecule-1; JAK2,janus kinase 2;MEK, MAPK/ERK kinase;mTOR, mammalian/mechanistic target of rapamycin;MMP, matrix metallopeptidase;MAPK,mitogen-activated protein kinases;RAF, mitogen activated protein kinase;RAS, mitogen activated protein kinase;mAChRs, muscarinic acetylcholine receptors;NK-1R, neurokinin-1 receptor; NGF, nerve growth factor;nAChR, nicotinic acetylcholine receptor;NE, norepinephrine;NF-kB, nuclear factor-kappa B;PLC, phospholipase C; PI3K, phosphoinositide 3-kinase;PKA, protein kinase A;PKC, protein kinase C;RET, proto-oncogene;AKT, serine/threonine kinase or protein kinase B;STAT3,signal transducer and activator of transcription 3; SP,substance P;TrkA,tropomyosin related kinase A

Tumor innervation influences metastasis as the ingrown nerve endings release neurotransmitters (such as norepinephrine, dopamine and substance P), which enhance metastatic spread [102]. To date, several neurotransmitters and neuropeptides involved in tumor metastasis have been identified (Table 1 and Fig. 1). In fact, several cancer cells express receptors for a number of neuropeptides and neurotransmitters, like norepinephrine, epinephrine, dopamine, GABA, acetylcholine, SP and NPY which have stimulatory effects on migration of cancer cells [103–112].

**Table 1:** Neurotransmitters influencing tumor metastasis

Neurotransmitters	Receptor	Type of cancer	Model	Mechanism/pathway	Ref.
NE	β2-AR	Pancreatic cancer	CFPAC1, MiaPaCa2 Panc1, and IMIM-PC2 cells	NE treatment reduces migratory activity of pancreatic cancer cells. NE mediates inhibitory effect via imbalanced activation of PKC/PLC signaling pathway → to activation of anti-migratory cAMP/PKA signalling.	[155]
		Prostate cancer	Subcutaneous injection of PC-3 cells in BALB/c nude mice	↑ NE leads to lumbar lymph node metastasis in an animal model.	[156, 157]
DA	DR1 & DR5	HCC	Tumor and non-tumor adjacent tissues from patients; LM3, Huh7 and SNU449 cells; subcutaneous injection of LM3 cells in BALB/c nude mice	DR5 is upregulated in tumor tissue and DR1 is upregulated in non-tumor human tissues. Dopamine ↑ cell proliferation in SNU449 cells. Administration of DR antagonist (thioridazine) inhibits cell proliferation in vitro and *in* and cell migration through EMT → ↓ tumor metastasis	[120]
GABA	GABA_A_	HCC	Human primary and adjacent non-tumor tissues, and Orthotopic inoculation of SMMC-7721 cells into the liver of BALB/c nude mice	GABA_A_receptor subunit ε1 expression is lower in human HCC tissues than in non-tumor liver tissues. GABA inhibits invasion and migration of human liver cancer cells in vitro*.* In mice, inoculation of SMMC-7721 cells pretreated with GABA ↓ tumor metastasis.	[128]
	GABA_B_		PLC/PRF/5 and Huh cells	Administration of GABA_B_ agonist (baclofen) ↓ cell migration associated with ↓ in intracellular cAMP levels.	[132]
		Breast cancer	Human tissues, 4 T1 and MCF-7 cells	Administration of GABA_B_ agonist (baclofen) promotes invasion and migration of breast cancer cells in vitro and metastasis in vivo via ERK_1/2_ and MMP-2signaling pathway.	[107]
		Prostate cancer	Human prostate and lymph node tissues, C4–2 cells	↑ Expression of GABA → cell invasion in vitro and lymph node metastasis in patients mediated by activation of MMPs signalling.	[158]
		HCC	Human primary and adjacent non-tumor tissues	The mRNA levels of GABA_B_ R1.2 and GABA_B_ R1.4 are higher in HCC tissues than in non-tumor liver tissues	[128]
ACh	AR	HCC	SNU-449 cells	ACh activates AR receptors → ↑ invasion and migration of SNU-449 cells via activation of AKT and STAT3 signaling pathways.	[133]
	α7-nAChR	Pancreatic cancer	CD18/HPAF, Capan1, FG/Colo357 cells in vitro and orthotopically implanted CD18/HPAF cells in immunodeficient mice	Nicotine treatment stimulates the expression of α7-nAChR and MUC4 in vitro*.* In the in vivo model, exposure to low and high cigarette smoking increases the tumor metastasis and MUC4 expression compared to sham controls. Nicotine induces tumor metastasis by upregulating MUC4 via α7-nAChR-mediated JAK2/STAT3 signaling in collaboration with Ras/Raf/MEK/ERK_1/2_ signalling pathway.	[135]
		Lung cancer	Line 1 cells in vitro, and subcutaneous injection of Line 1 cells in BALB/c mice	Intraperitoneal injection of nicotine ↑ tumor growth and metastasis through change in gene expression via nAChR signalling pathway.	[159]
	nAChR β2	Lung cancer	B16 cells intravenous injection in C57BL/6 mice	↑ Nicotine exposure → activation of nAChR β2 on NK cells mediates metastasis	[160]
	α9-nAChR	Breast cancer	MDA-MB-231 and MCF-7 cells	Nicotine treatment enhances the migratory abilities of both cells by activating α9-nAChR through elevated expression of EMT markers	[134]
	mAChR	Colon cancer	Hh508 and SNU-C4 cells	Administration of muscarinic inhibitor (atropine) → ↓ cell invasion and migration. ACh binding to M3R mediates cell migration via the activation of post-ERBB1, ERK and PI3K-dependent RhoA pathway.	[138, 139]
		NSCLC	Human tissues, micA549, PC9, SPC-A1, GLC82, L78 and HLF cells	M3R expression correlates with clinical stage and poor survival in patients. M3R stimulation by ACh enhances in vitro cell invasion and migration via PI3K/AKt pathway.	[136, 137]
		Prostate cancer	Human tissues, Hi-Myc transgenic mice-bearing PC-3	Presences of cholinergic nerve fibers associate with poor clinical outcome in human patients. Pharmacological blockade or genetic disruption of the M1R inhibit metastasis leading to improved survival of the mice	[93]
SP	NK-1R	Pancreatic cancer	MiaPaCa-2, BxPC-3, CFPAC-1, HAPC, Panc-1, and SW1990 cells	Binding of SP to NK-1R promotes cell invasion and migratory potential which is mediated by expression of MMP-2. SP also increases cell migration and neurite outgrowth toward DRG demonstrating important role in metastasis and PNI.	[146, 161]
NPY		Ewing sarcoma	Human serum, SCID/beige mice bearing SK-ES1 cells	Enhanced level of systemic NPY associate with metastatic tumors. In the xenograft model, NPY expression associate with bone metastases.	[149, 150]
	Y5	Breast cancer	4 T1 cell line	NPY mediates metastatic effect via the activation of Y5 receptor.	[148]
Neurotensin	NTSR1	Breast cancer	Human tissues	The expression of NTSR1 associates with lymph node metastasis.	[151]

*Ach* acetylcholine, *AR* androgen receptor, *β2-AR* β_2_-adrenergic receptor, *cAMP* cyclic adenosine monophosphate, *DA* dopamine, *DR* dopamine receptor, *DRG* dorsal root ganglia, *ERBB1* epidermal growth factor receptor 1, *EMT* epithelial–mesenchymal transition, *ERK*_*1/2*_ extracellular signal-regulated kinase, *GABA* gamma-aminobutyric acid, *GABA*_*A&B*_ gamma-aminobutyric acid receptor _A&B_, *HCC*, hepatocellular carcinoma, *JAK2* janus kinase 2, *MEK* MAPK/ERK kinase, *MMP* matrix metallopeptidase, *RAF* mitogen activated protein kinase, *RAS* mitogen activated protein kinase, *MUC4* mucin 4, *mAChRs* muscarinic acetylcholine receptors, *M3R* muscarinic receptors 3, *NK* natural killer cells, *NK-1R* neurokinin-1 receptor, *nAChR* nicotinic acetylcholine receptor, *NSCLC* non-small cell lung cancer, *NE* norepinephrine, *PNI* perineural invasion, *PLC* phospholipase C, *PI3K* phosphoinositide 3-kinase, *PKA* protein kinase A, *PKC* protein kinase C, *RhoA* Ras homolog gene family member A, *AKT* serine/threonine kinase or protein kinase B, *STAT3* signal transducer and activator of transcription 3, *SP* substance P

### Catecholamines

The increased expression of β-adrenergic receptor for catecholamines is associated with poor prognosis in breast cancer [113]. Stress stimulation leads to macrophage infiltration to the tumor site which activates β-adrenergic signaling pathways leading to increased metastasis in an orthotopic breast cancer model in BALB/c mice [57]. In this model, administration of β-adrenergic antagonist, propranolol, decreases breast cancer metastasis [57]. Similarly, the use of β-blockers in breast cancer patients inhibits metastasis and disease recurrence as well as improving survival of patients [113, 114]. In ovarian cancer patients, the grade and stage of tumors correlate with higher tumor norepinephrine levels associated with stress [115]. In an orthotopic mouse model of ovarian cancer, chronic stress elevates tumor noradrenaline levels and increases the aggressiveness of tumor growth [49]. In prostate cancer C42 xenografts in nude mice and Hi-Myc mice with prostate cancer, plasma adrenaline promotes carcinogenesis via β_2_ adrenergic receptor/protein kinase A/BCL2-associated death protein anti-apoptotic signaling pathway [116]. Hence, stimulation of catecholamines plays a major role in activation of signals for breast cancer metastasis. Therefore, inhibition of the sympathetic nervous system signaling pathways with β-blockers holds great promise in preventing metastasis of various tumors including breast cancer. On the other hand, involvement of α-adrenergic receptors in cancer metastasis is not well understood. In the murine model of metastatic mammary adenocarcinoma induced by 4 T1 cells in BALB/c mice, activation of α_2_-adrenergic receptors increases tumor growth rate and the number of metastasis [117]. In contrast, blockade of α-adrenergic receptors in the absence of stress increases distant metastasis in the orthotopic model of mammary adenocarcinoma induced by MDA-MB-231HM cell line in nude mice [118].

The role of dopamine in cancer metastasis is not clear. Low levels of dopamine have been reported in stressed mice with ovarian carcinoma [119]. In contrary, in hepatocellular carcinoma (HCC) patients dopamine levels are elevated in the blood samples compared to healthy individuals [120]. Moreover, enzymes such as monoamine oxidase A (MAOA) degrading catecholamines and serotonin [121] may also play an important role in influencing cancer metastasis [122–124]. Studies have demonstrated that MAOA expression is decreased in HCC patients; it suppresses HCC cell metastasis by inhibiting adrenergic and epidermal growth factor receptor (EGFR) signaling pathways [125]. Inhibition of MAOA stimulates malignant behavior in MDA-MB-231 breast cancer cells [126]. On the other hand, high expression of MAOA in human tissues correlates with poor prognostic in prostate cancer patients and increased tumor metastasis in xenograft mouse model of prostate cancer via HIF1-α/VEGF-A/FOXO1/TWIST1 signaling pathway [124]. These limited studies on the role of MAOA in cancer metastasis are controversial.

### γ-Aminobutyric acid (GABA)

Plays a role in cancer metastasis via activation of ionotropic (GABA_A_) and metabotropic (GABA_B_) receptors [127]. It has been demonstrated that GABA mediates its inhibitory effect through GABA_A_ receptor. For example, HCC cell lines and human adjacent non-tumor liver tissues, express GABA_A_ receptor. GABA inhibits HCC cell migration through the activation of GABA_A_ receptor [128]. However, there are studies demonstrating that GABA_A_ receptor enhances metastasis. The activation of GABA_A_ receptors upregulates brain metastasis of breast cancer patients [129]. Expression of the GABA_A_ receptor subunit, Gabra3, which is normally not present in breast epithelial cells, is increased in human metastatic breast cancer which correlated with poorer patients survival [108]. Gabra3 overexpression promotes migration and metastasis of breast cancer cells via activating serine/threonine kinase or protein kinase B (AKT) signaling pathway demonstrated in a mouse orthotopic model induced by MCF7 and MDA-MB-436 breast cancer cell lines [108]. Mechanistically, the activation of AKT signaling pathway enhances metastasis via downstream molecules such as focal adhesion kinase and MMPs [130, 131]. Therefore, it could be speculated that the effect of GABA_A_ receptor depends on the activated downstream molecules and signalling pathways. Murine (4 T1) and human (MCF7) breast cancer cell lines and human breast cancer tissues express GABA_B_ receptor [107]. In mice, GABA_B_ receptor mediates 4 T1 cell invasion and pulmonary metastasis via ERK_1/2_ signaling [107]. GABA_B_ activation inhibits migration of PLC/PRF/5 and Huh 7 malignant hepatocyte cell lines in vitro [132].

### Acetylcholine (ACh)

Plays a functional role in cellular proliferation, differentiation and apoptosis. In HCC, the release of ACh acting on androgen receptor promotes SNU-449 cell invasion and migration via activation of AKT and signal transducer and activator of transcription 3 (STAT3) signaling pathways [133]. Nicotine stimulation of nicotinic acetylcholine receptor (nAChRs) enhances SW620 and LOVO colorectal cancer cell invasion and metastasis in vitro via the activation of p38 mitogen-activated protein kinases (MAPK) signaling pathway [112]. Similarly, nicotine pretreatment stimulates the activation of α9-nAChR which mediates MCF-7 and MDA-MB-231 breast cancer cell migration via the expression of epithelial mesenchymal transition markers [134]. Furthermore, implantation of CD18/HPAF pancreatic cancer cells into immuno-deficient mice, demonstrates that nicotine treatment activates α7-nAChR and mediates tumor metastasis via Janus kinase 2 (JAK2)/STAT3 signaling in synergy with mitogen activated protein kinase (Ras/Raf/MEK/ERK_1/2_) signalling pathway [135]. ACh promoted cancer metastasis and associate with poor clinical outcomes in prostate adenocarcinoma via M1R; and pharmacological blockade or genetic disruption of the M1R inhibit tumor invasion and metastasis leading to improved survival of the mice-bearing PC-3 prostate tumor xenografts [93]. In addition, ACh acting on M3 muscarinic receptor (M3R) associates with metastasis and low survival rate of NSCLC patients [136]. M3R activation increases invasion and migration of NSCLC cells and increased release of interleukin (IL)-8 via the activation of EGFR/PI3K/AKT pathway [137]. In human SNU-C4 and H508 colon cancer cell lines, administration of muscarinic receptor inhibitor, atropine, abolished SNU-C4 cell migration, however, H508 cell migration requires the activation of MMP7 [138, 139].

### Neuropeptides

Expression of SP is shown to exert functional effects on small cell lung cancer [140], pancreatic [141], colon [142], prostate [143, 144] and breast cancer [145] cells. SP acting on neurokinin-1 (NK-1) receptors enhances pancreatic cancer cell migration and perineural invasion to the dorsal root ganglia (DRG) mediated by MMP-2 demonstrating its essential role in metastasis [146]. Enhanced expression of SP correlated with lymph node metastasis and poor prognosis in colorectal cancer patients [142]. NPY modulates cell proliferation, differentiation and survival via acting on its G protein-coupled receptors designated Y1R–Y5R leading to the development of metastasis [147, 148]. High levels of systemic NPY associates with metastatic tumors as noted in Ewing sarcoma patients [149]. Similarly, in the SK-ES1 xenograft model, elevated levels of NPY associates with bone invasion and metastases [150]. NPY mediates 4 T1 cell proliferation and migration via the activation of NPY Y5 receptor [148]. Neurotensin mediates metastasis by binding to neurotensin receptors 1 (NTSR1). In breast cancer, the expression of NTSR1 correlates with lymph node metastasis [151]. These studies demonstrate the important role of neuropeptide signaling in cancer metastasis.

## Concluding remarks and future directions

Metastasis continues to be the main cause of cancer-related death. Although genetic compartments that influence metastasis have been identified, there are still needs to conduct comprehensive evaluation of the factors that contribute to cancer metastasis. This review demonstrates that the nervous system influences cancer metastasis through the release of neurotransmitters and neuropeptides leading to metastasis. However, sensory nerve fibres have been given less attention. Sensory stimuli activate pain transmission pathways which result in acute or chronic pain depending on the intensity and the nature of the stimulus [152, 153]. Cancer-related pain is linked to accelerating cancer progression and metastasis. Sensory nerves can innervate primary tumors and metastases, thus contributing to tumor-associated pain as demonstrated in pancreatic [61] and prostate cancer [154]. Therefore, a possible involvement of sensory fibers in tumor progression and metastasis, although not well demonstrated at this stage, cannot be excluded.

In conclusion, cancer cells can transduce neurotransmitter-mediated intracellular signaling pathways which lead to their activation, growth and metastasis. The findings reported here are primarily done in cancer cell lines and animal models. Therefore, better understanding the interaction between these signaling molecules and tumor cells in human cancers would enhance our knowledge on pathways promoting cancer metastasis.
